# Treatment of Dysentery

**Published:** 1852-11

**Authors:** 


*Treatment of Dysentery.* By GEO. S. UPSHUR, A. M., M. D., of Norfolk, Va.

We quote the following from an article on Dysentery, by Dr. Upshur, in the Philadelphia Med. Examiner.—ED. BUFFALO MED. JOUR.

No agents have been more overrated in the treatment of dysentery than mercurials. The combination of calomel, opium and ipecac., is the *alpha* and *omega* of therapeutics in this disease, with a very large and respectable body of physicians; and such is the testimony adduced in its favor that I doubt not beneficial results have *followed* its use in particular cases. I am disposed, however, to attribute the benefits to the *opium and ipecac. only*, and am satisfied from careful and ample observation, that they would have accomplished much more good without the calomel. It may be true that in the dysentery of tropical climates, where torpor of the liver is usually a complication, mercury is sometimes administered with benefit, but in the ordinary dysentery of this country I am persuaded it does more harm than good. My own observation in this regard is corroborated by some of the best authorities in medicine.

Dr. Cheyne, in 3d vol. Dublin Hospital Reports, says: "Mercury could not be depended upon, and did not relieve in numerous instances when the mouth was affected, and sometimes seemed to increase the disease."

M. Twining, who saw a vast amount of the disease in the General Hospital at Calcutta, says: "I speak without hesitation on this subject, from having too often seen the fallacy of trusting to the effects of calomel for the cure of severe acute dysentery, and having tried that medicine extensively in every form of the disease."*

Dr. Mackintosh, (*Practice of Physic*, vol. i. p. 366, 1837,) says: "My own experience in this country, as well as within the tropics, enables me to confirm the statement of Dr. Cheyne and M. Twining."

Dr. C. A. Lee, in a note to the art. *Dysentery*, in Copland's Dictionary, says: "With respect to the use of mercury in this disease, we believe that it generally proves injurious."

Dr. Dunglison says that the common remark, that if the disease proves obstinate it will yield, provided we can only 'touch the mouth' with a mercurial, does not accord with his experience. He has seen many cases in which the effect of mercury has been induced on the system, and, nevertheless, the disease has proceeded to a fatal termination. (*Practice of Medicine*, vol. i. p. 114.)

Dr. Batchelder, in the N. Y. Journal of Medicine for July, 1851, confirms

---

* Vide Diseases of Bengal, by W. Twining, Esq., p. 40.

these statements by adding his own reliable testimony against the calomel practice.

But it is useless to multiply authorities upon this subject. I will add a case or two in illustration from my case-book:

The cases are omitted.—Ed. Buffalo Jour.

*Saline Cathartics.*—I have prescribed a combination of Epsom and Rochelle Salts, ʒij. of each, after general blood-letting, with the happiest effects in many cases of acute dysentery—and am disposed to think very favorably of the practice. I think it is particularly applicable to cases occurring in robust persons where the tenesmus is very troublesome. Frequently have I seen this symptom entirely relieved after the copious watery evacuations produced by the saline.

*Acids.*—The nitrous acid mixture of Dr. Hope, (℞. Acid Nitrosi ʒi. Tr. Opii gtt. xl.; Aquæ Camph. ℥viii. M.) which was introduced to the profession in this country through the columns of this Journal, by Dr. C. D. Meigs, in 1838, is justly considered by those who have used it freely, a remedy of great value in dysentery. Among all the good things which Dr. Meigs has done for his brethren, he never did a better than calling their attention to this remedy. In my hands it has rarely failed to accomplish every thing that was desired. It is not a specific, but if fairly tried I think will less often bring disappointment than any other single remedy in this frequently intractable affection. Great care should be taken not to administer it in too small doses, an adult should not take less than ℥ii. at a time, and it should be continued at intervals of three or four hours until about eight doses have been given.

*Opium and Acetate of Lead.*—Since the publication of Dr. Batchelder's valuable article, before referred to, I have given the acetate of lead and opium a fair trial, and am prepared to speak as favorably of the combination as Dr. B. has done. My experience with it has been in those cases chiefly where Hope's mixture failed. I usually give as the first dose, one grain of opium with four of the acetate, and then half a grain of the one with two grains of the other, *immediately after each evacuation,* no matter if the bowels are moved every five minutes. Usually but a few doses are taken before relief is obtained.

